# Effects of Two Nights of Severe vs. Mild Sleep Restriction on Vertical Jump Performance in Physically Active Female Students

**DOI:** 10.3390/life16030443

**Published:** 2026-03-09

**Authors:** Andrija Miksa, Antonio Martinko, Luka Milanovic, Marin Dadic, Ivan Belcic

**Affiliations:** 1 Faculty of Kinesiology, University of Zagreb, 10 000 Zagreb, Croatia; 2 Faculty of Chemical Engineering and Technology, University of Zagreb, 10 000 Zagreb, Croatia

**Keywords:** jump performance, sleep restriction, sports performance, sleep quality

## Abstract

Partial sleep deprivation is common in sports, particularly before competitions. This study examined whether two nights of severe sleep restriction (<4 h/night), compared with mild sleep restriction (control), are associated with changes in SJ and CMJ in physically active female students. Twenty-three female students (*n* = 12 experimental; *n* = 11 control) were randomly assigned to their respective groups. The experimental group underwent two nights of severe sleep restriction (<4 h/night), while the control group experienced mild sleep restriction. Differences between groups were analyzed using Quade’s nonparametric ANCOVA (sleep duration as covariate), and within-group pre–post changes were evaluated using paired-samples *t*-tests. No significant differences were found between groups after two nights in CMJ (*p* = 0.92) or SJ (*p* = 0.73) performance. Within the experimental group, SJ performance significantly decreased from the initial to the final assessment (*p* = 0.02), with a large effect size (d = −0.81). CMJ performance in the experimental group showed a non-significant decrease with a moderate effect size (d = −0.45). No significant differences or notable effect sizes were found in the control group (d = 0.01 to 0.23). Within-group results suggest that SJ decreased after severe sleep restriction, while CMJ changes were smaller and not statistically significant; between-group comparisons were not significant, and results appear sensitive to the analytical approach. These exploratory findings suggest that monitoring sleep before power-related tasks may be warranted. Coaches may consider monitoring sleep duration before high-intensity power training, as two nights of severe sleep restriction may be associated with reduced SJ performance.

## 1. Introduction

Sleep deprivation is common in sports [1,2]. Studies indicate that 15–30% of athletes experience sleep problems, particularly before major competitions [1,3], a finding corroborated by several other investigations [4,5,6]. The primary causes of poor sleep include tension, anxiety, and pre-competition rumination, which may be mitigated through various behavioral, nutritional, and environmental strategies [7,8]. In the athletic population, partial sleep deprivation can also result from time zone changes associated with travel demands [9]. Specifically, sleep quality deteriorates when individuals attempt to sleep outside their circadian sleep phase [9]. Apart from the night before a competition, which may independently contribute to poor sleep quality [10], even routine training schedules can induce some form of partial sleep deprivation throughout the week. For example, one study [11] demonstrated that basketball players’ performance is impaired in the morning compared to the afternoon, primarily due to insufficient sleep caused by morning training sessions. Recent evidence suggests that female athletes may be more vulnerable to the effects of extended wakefulness and circadian misalignment compared to males [12].

Power output in the vertical plane, which is related to jumping ability, is critical in many sports. Research highlights that vertical jumping ability is fundamental to the performance of several key skills across various sports, such as the jump shot in handball, spiking in volleyball, heading in soccer, high jump and long jump in track and field, etc., which can influence outcomes in these sports [13,14,15,16,17,18,19]. Authors in several studies found that partial sleep deprivation can have a significant influence on sports performance [20,21,22].

Studies examining the effects of various forms of sleep deprivation on jump performance have yielded varying results. It appears that the negative impact on jump performance increases with the duration of sleep restriction. Some research has demonstrated a detrimental effect of complete sleep deprivation on jump performance [23,24], while other studies have not observed significant differences [25,26]. Given that complete sleep deprivation is rare in real-life scenarios and partial deprivation is more common, it is crucial to assess the effects of partial sleep deprivation on jump performance. One study [27] investigated the impact of sleep deprivation on countermovement jump (CMJ) performance, finding that complete sleep deprivation significantly decreased CMJ performance by 11.4%. In contrast, partial sleep deprivation impaired performance by 5.2%, though the result was not statistically significant. Another study [28] reported that partial sleep deprivation lasting three days (approximately 4 h of sleep per night) significantly reduced maximum jump height in elite athletes. These findings suggest that partial sleep deprivation adversely affects jump height, yet the precise context, the extent of deprivation required, and the specific population affected remain unclear due to the diversity of study participants.

The majority of studies predominantly utilize male subjects, leaving the specific effects of partial sleep deprivation on jump performance in women less understood. Evidence indicates that the squat jump (SJ) and countermovement jump (CMJ) on a force platform are highly reliable and valid assessments for evaluating jump performance and neuromuscular fatigue [29,30]. Sleep deprivation is known to cause both physical and mental fatigue [31], necessitating the use of highly sensitive tests to detect subtle changes in performance due to fatigue. This study aims to investigate the impact of two nights of partial sleep deprivation on the jump performance of young, physically active women, with the hypothesis that two nights of severe sleep restriction (with less than 4 h of sleep) will reduce CMJ and SJ performance in young physically active women.

## 2. Materials and Methods

### 2.1. Subjects

Twenty-three first-year female students from the Faculty of Kinesiology, University of Zagreb, with an average age of 19.44 ± 0.75 years participated in the study. All participants were highly physically active, engaging in some form of physical activity or training 5–7 times per week without control over the intensity or type of training. This information was self-reported, providing valuable context for result generalization, as these individuals are probably more akin to athletes than to average students. Inclusion criteria required participants to be normal sleepers (PSQI ≤ 5), based on the Pittsburgh Sleep Quality Index (PSQI). Habitual sleep duration of 7–9 h per night was self-reported. Potential subjects that did not meet this criterion were excluded from the study. All participants needed to possess a smartphone capable of installing the application used in this study. Female students meeting these criteria were included after providing written informed consent. All participants volunteered for the experiment, which adhered to the ethical principles outlined by the Code of Ethics of the Faculty of Kinesiology, University of Zagreb (Ethics Committee protocol code—17/2024). Subjects were randomly divided into an experimental group that slept less than 4 h for 2 nights in a row and a control group that followed the study protocol without severe restriction (mild sleep restriction during the study).

### 2.2. Description of Testing Protocols

Subjects performed the initial testing procedure after a night of habitual sleep. Prior to this, randomization into groups was conducted. As previously mentioned, inclusion in this study required meeting the criteria of being a “normal sleeper” according to [32], assessed using the Pittsburgh Sleep Quality Index (PSQI) [33]. The PSQI was utilized because it evaluates sleep quality and disturbances over a one-month period. Subjects with a PSQI score above 5, indicating poor sleep quality, were excluded from participation. All eligible subjects reported habitual sleep durations between 7 and 9 h. Participants were required to maintain their usual sleep patterns, with 7–9 h of sleep per night, for the four nights preceding the initial testing.

The experiment spanned three days and two nights, designed to simulate partial sleep deprivation over two consecutive days (4 or fewer hours of sleep per night) and to investigate a potential relationship between partial sleep deprivation (PSD) and jump performance. Due to technical difficulties, data collection after the first night of PSD was not possible. Consequently, the squat jump (SJ) and countermovement jump (CMJ) tests were only conducted after the second night of PSD. Testing occurred at two time points: an initial test and a final test, with no follow-up. To ensure consistency, testing was conducted at the same time of day for both time points. Subjects were tested in small, consistent groups, and all procedures were performed by the same expert. Tests were administered in the same sequence for each group.

During the experiment, the experimental group experienced partial sleep deprivation, sleeping four or fewer hours per night (Table 1). The control group experienced mild sleep restriction during the protocol. Sleep duration was monitored using the Sleep Time mobile application developed by Azumio (Azumio, Inc., Palo Alto, CA, USA). Mobile applications, such as Sleep Time, have been shown to have similar sensitivity in detecting sleep and wakefulness as actigraphy [34,35]. When compared to polysomnography, the Sleep Time app demonstrated high sensitivity but low specificity (89.9% and 50%, respectively) [36]. The application was selected for its ease of use, popularity, and free availability on both iOS and Android platforms, which facilitated participant adherence and duration tracking rather than clinical sleep architecture. The application did not provide objective sleep architecture, sleep stages, or validated sleep quality measures, as it was used primarily to support adherence and estimate time asleep. Also, it is important to note that daytime naps were not recorded. In addition to using the app, subjects kept a sleep diary and recorded their sleep and wake times. Subjects also communicated with researchers before and after sleep via text messages (e.g., “Now, I am going to bed” or “I have woken up”) to ensure compliance. While the app did not provide accurate sleep quality data, all measures were taken to monitor and verify sleep duration.

Subjects slept in their habitual environments and were prohibited from consuming alcohol, nicotine, caffeine, or other central nervous system stimulants for eight hours prior to each test. The last meal had to be consumed at least eight hours before testing. Although there was no continuous control over participants’ daytime activities, they were informed that researchers might contact them randomly to enhance adherence to the sleep deprivation protocol. Testing was conducted at the Diagnostic Center of the Faculty of Kinesiology, University of Zagreb, using the QUATTRO JUMP force measurement platform (Kistler, Switzerland). The system is built on piezoelectric sensor technology, which is known for its high sensitivity and wide measuring range: (Fz): 0 to 10 kN/linearity: <±0.5%FSO (full scale output), hysteresis: <1%FSO/sampling rate: 500 Hz/resolution: 14-bit data acquisition, providing a resolution of approximately 0.2 N to 1 N per bit depending on the range setting.

Upon arrival, subjects underwent a standardized warm-up consisting of a light 3 min run, dynamic leg muscle stretching, 10 squats, 5 jumps without preparation, and 5 jumps with preparation. Following a 3 min rest, subjects performed three repetitions of the squat jump (SJ) followed by three repetitions of the countermovement jump (CMJ), with a 1 min pause between each jump type.

### 2.3. Description of Measuring Instruments and Variables

#### 2.3.1. Squat Jump (SJ)

After positioning on the platform with both legs, subjects adopted a squat position with their hands on their hips. The angle between the upper and lower legs was visually inspected to ensure approximately 90 degrees of flexion. Once properly positioned, subjects performed a maximal vertical jump from this position upon the examiner’s signal. Participants were instructed not to squat deeper upon receiving the signal to avoid pre-stretching the muscles, thereby minimizing the influence of the stretch-shortening cycle (SSC). Each subject performed the jump three times, with the best result being recorded. If a jump was performed with prior preparation involving the SSC, it was repeated.

#### 2.3.2. Countermovement Jump (CMJ)

Proper positioning on the platform for this test was the same as for the previous squat jump (SJ) test. Subjects stood in an upright position before initiating movement. Upon the examiner’s signal, subjects performed a preliminary squatting motion by bending at the hips and knees, followed by a maximal vertical jump through the full extension of all leg joints. The depth of the squat to the half-squat position was self-selected by each subject, but consistency across repetitions was required. Each subject performed the jump three times, and the best result from these attempts was recorded. The best attempt from the three trials was used for analysis. The jump technique was standardized through verbal instructions and visual monitoring; however, kinetic variables and force–time curve characteristics were not analyzed to objectively verify execution consistency.

### 2.4. Statistical Analyses

Data processing was performed using Microsoft Excel and SPSS (version 30.0). The required sample size was calculated with G*Power software (version 3.1.9.7, Düsseldorf, Germany) for ANCOVA (fixed effects, main effects, and interactions), based on an effect size (ES) of 0.65, an alpha level of 0.05, and a power (1-β) of 0.80. The required total sample size was 21, with three additional participants included to account for potential withdrawal from the study.

Initially, all data were tested for normality using the Shapiro–Wilk test. For normally distributed data, descriptive statistics were calculated and presented as mean, minimum values (MIN), maximum values (MAX), and standard deviations (SDs). An F-test was conducted to assess the homogeneity of variances between the two groups. Linearity between covariates and dependent variables was determined using scatterplots. Because linearity was not found in one measurement, differences between the experimental and control groups at each time point were analyzed using Quade’s nonparametric ANCOVA, while a paired samples *t*-test was used to evaluate differences between the initial and final measurements for both groups. Given that the duration of sleep varied among participants and groups, sleep duration over both nights was included as a covariate in Quade’s ANCOVA model.

## 3. Results

The Shapiro–Wilk test confirmed normal distribution for all variables (*p* > 0.05). The F-test indicated that the two groups had equal variances (*p* > 0.05). Linearity between the covariate (sleep duration) and at least one dependent variable was not observed; for that reason, Quade’s nonparametric ANCOVA was performed.

**Figure 1:**
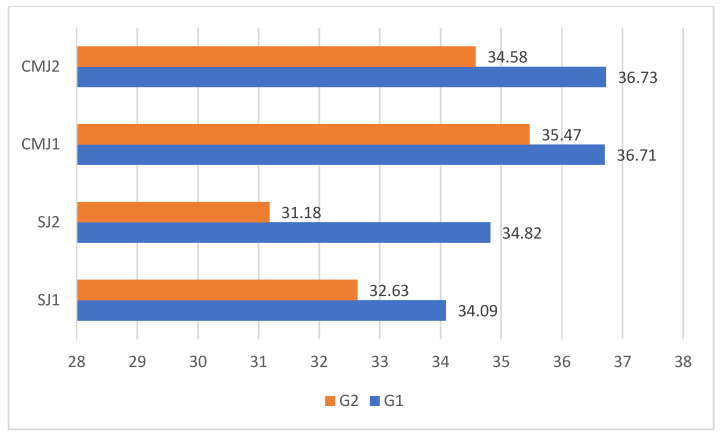
Pre–post change in SJ and CMJ by group.

Table 1 and Table 2 show the basic statistical indicators for the experimental and control groups in all variables. Table 1 shows the sleep duration variable on the first and second day of the protocol for the experimental and control groups. Figure 1 shows the pre–post changes in SJ and CMJ in the control and experimental groups. Table 3 shows the results of the paired samples *t*-test for the experimental and control groups. SJ and CMJ mean differences increased slightly in the control group (0.73 and 0.02 for SJ and CMJ) and decreased in the experimental group (−1.44 and −0.88 for SJ and CMJ). Significance was reached only for the SJ variable in the experimental group. The effect size for the experimental group was moderate to large (0.45–0.81) for CMJ and SJ, respectively, whereas the control group exhibited negligible effect sizes (0.23–0.01). In Table 4, Quade’s test identified no significant differences between the experimental and control groups. A sensitivity analysis examining the correlation between sleep duration and jump performance revealed no significant associations (Table 5).

## 4. Discussion

Quade’s test identified no significant differences between the experimental and control groups. These findings should be considered exploratory and not interpreted as evidence of a group effect. A significant difference was observed between the initial and final measurements in the SJ variable in the experimental group. The effect size for the experimental group was moderate to large (0.45–0.81) for CMJ and SJ, respectively, whereas the control group exhibited negligible effect sizes. The following section will analyze the potential causes of these findings.

Within-group changes suggest that SJ may decline after severe sleep restriction; however, the between-group comparison did not detect significant differences. This aligns with previous research indicating that sleep deprivation (partial or complete) adversely affects jump height [23,26,27,37,38]. In other research, authors [27] demonstrated that elite athletes experiencing three consecutive nights of partial sleep deprivation (approximately four hours of sleep per night) exhibited a decrease in maximum jump height. Consistent with prior studies, the present research found that SJ height decreased by 4.4%, while CMJ height declined by 2.5%. In contrast to [26], where CMJ height was reduced by 5.2%, the decrease observed in this study was approximately half of that. This discrepancy may be attributed to differences in participant characteristics, as the sample in [27] consisted of physically active males. In a similar study [39], a 1.5% reduction in jump height was reported following four hours of partial sleep deprivation in athletes. Their study utilized drop jumps, which involve the storage of elastic energy within the muscle–tendon complex, potentially mitigating performance declines compared to the current study. Notably, in this research, the reduction in CMJ height was twice as small as that observed in SJ, suggesting distinct underlying mechanisms. The prior literature suggests a dose–response relationship between the degree of sleep restriction and performance decrements, and our study could not test this directly because measurements were not obtained after the first night, and the between-group comparison was not significant. This is consistent with recent meta-analyses showing that explosive power is one of the domains most consistently impaired by early-night sleep restriction [40]. Motivation, expectancy effects, and familiarization may have influenced performance, which could partly explain the non-significant between-group findings despite within-group changes. This should be considered when interpreting the magnitude and direction of SJ and CMJ changes.

The precise reasons for these differences remain unclear, and direct comparisons with other studies are limited due to the scarcity of research and differences in study protocols. One potential explanation for the greater impairment in SJ performance is the biomechanical differences between SJ and CMJ. Specifically, during CMJ execution, a greater distance is covered in the maximal active state, facilitating a more rapid reduction in muscle slack compared to SJ [36]. A certain amount of muscle and tendon slack must be overcome to initiate movement, whereas in CMJ, there is less or no slack because of the active state [36]. Research shows that when performing SJ, the most important determinant of performance is peak force at the beginning of the movement, which is recorded before the initiation of the movement itself [39]. In contrast to the SJ, it was found that the amount of force after 50 milliseconds was crucial for better CMJ performance, i.e., during the second part of the movement [41]. Certain capacities may fall earlier under the influence of partial sleep deprivation. Given that this rapid force production relies on high levels of muscle stimulation, it is plausible that sleep deprivation disrupts this capability. Consequently, fatigue resulting from partial sleep deprivation may reduce participants’ capacity for rapid force production, thereby disproportionately affecting SJ performance, as CMJ allows more time for force generation. Because only the within-group SJ reached significance and the between-group test was null, mechanistic interpretations should be considered tentative.

These findings should be interpreted with caution as the sample size was relatively small, limiting statistical power, and a larger sample could yield different results. Quade’s test suggested that actual sleep duration had no significant effect on jump performance, indicating no clear dose–response relationship between the extent of sleep deprivation and performance declines. Given the minimal variability in sleep duration among participants, such an outcome was expected. These findings indicate that interpretation is sensitive to the analytical approach (within-group vs. between-group comparisons). The sensitivity analysis did not provide consistent evidence of an association between total sleep duration and changes in SJ or CMJ, which may suggest that the underlying effect of sleep restriction on vertical jump performance is small in this sample. It is important to note that the key findings discussed are derived from within-group changes only, as no statistically significant differences were observed between the groups. This highlights that the direction of the main conclusion varies depending on the analytical approach used (within-group vs. between-group), suggesting that the underlying effect of the sleep restriction protocol on performance was weak.

The control group experienced mild sleep restriction, which reduced the contrast between groups and likely attenuated detectable between-group effects. Sleep duration was not uniform across all participants, as the control group averaged approximately 6.55 h of sleep on the second night, which constitutes mild sleep restriction. Consequently, this study should be interpreted as a comparison between severe (experimental) and mild (control) sleep restriction, rather than a comparison against a fully rested control group. A possible cause of less sleep, in addition to the aforementioned obligations, is potentially factors related to psychological pressure [4,8]. Given that the subjects were highly motivated and wanted to achieve the required amounts of sleep, this could have had the opposite effect because research has shown that high amounts of motivation and wanting to fall asleep create cognitive arousal and pressure, thus creating a physical and cognitive environment that prevents sleep [42,43].

Because no acclimatization session was provided, it is possible that some participants, due to the learning effect, increased performance from initial to final measurements, as seen by the variation in minimum and maximum values, especially in the control group, further limiting firm conclusions.

This study has some notable strengths and limitations that should be considered when interpreting the findings. A primary strength is its high validity, as the protocol simulates the real-world partial sleep deprivation of less than four hours, which is often experienced by athletes before competition. Also, one of the strengths of the study could be a gap in the literature by focusing on physically active females, a demographic less studied than males. Methodological rigor was supported using the reliable QUATTRO JUMP force platform, the Pittsburgh Sleep Quality Index to ensure participants were “normal sleepers”, and a clear process involving sleep diaries and text communication to monitor adherence. Regarding the strengths of the study, there are also several limitations, which include a relatively small sample size that may limit statistical power and not controlling the daytime physical activity. The methodology used to assess sleep duration was a mobile application, which lacks reliability compared to objective measures such as actigraphy [33,35]. The mobile application was used primarily to support adherence and estimate time asleep, as it is known that it cannot provide validated sleep architecture, sleep stages, or objective sleep quality measures comparable to actigraphy or polysomnography. Due to the absence of advanced equipment and with the aim of using the application not only as a tool to quantify sleep duration but to adhere to the protocol because they need to bring the data from the mobile app) and a supplement to sleep diary rather than a primary measurement tool, the authors believe its use positively influenced the study by increasing participant engagement. To reinforce adherence to the protocol, participants were required to send messages to the researchers before sleep and upon waking, ensuring additional verification of compliance. While these measures helped control sleep duration (or more accurately, time spent in bed) at night, a limitation remains as participants were not monitored during the day, leaving a lack of control for daytime naps and physical activities. The use of a mobile application for sleep tracking lacks the specificity of polysomnography, and the inability to collect data after the first night prevented the evaluation of cumulative dose–response effects. It is important to mention the results that the authors could not affect—the control group experienced a mild reduction in sleep duration during the study. Although jump height was measured, kinetic variables or force–time curve characteristics (e.g., peak force, impulse, rate of force development) were not analyzed to objectively confirm consistent jump execution between sessions. This limits the ability to determine whether observed changes reflect true neuromuscular alterations versus subtle technique differences. Menstrual cycle phase and hormonal status were not monitored or controlled. As hormonal fluctuations may influence sleep, perceived fatigue, and neuromuscular performance, this may have introduced additional variability in SJ and CMJ outcomes.

The practical implications of this study suggest that coaches should prioritize monitoring athlete sleep duration, particularly 48 h prior to high-intensity training or competition, as two nights of severe sleep restriction (<4 h/night) may be associated with reduced explosive power, particularly in tasks requiring rapid force production such as the squat jump. Coaches may recognize that while countermovement-based actions (CMJ) may be more resilient to short-term sleep loss due to a longer duration of force generation, static-start explosive movements (SJ) are highly sensitive to fatigue and may require training volume adjustments when athletes report poor sleep. To reduce these risks to a minimum, staff should implement sleep hygiene strategies and avoid scheduling late-night travel or early morning sessions that prevent athletes from obtaining adequate sleep before competitions or high-intensity training.

## 5. Conclusions

Within-group results suggest that SJ decreased after severe sleep restriction, while CMJ changes were smaller and not statistically significant; between-group comparisons were not significant. These findings should be interpreted cautiously, given the null between-group effects and the study’s limitations. Limited sleep measurement precision, lack of control over daytime activity, and the relatively small sample size limit the strength of the conclusions. The reasons for the larger change in SJ than CMJ remain speculative; one possible explanation is that severe sleep restriction may reduce rapid force production, which could disproportionately affect static-start jumps, such as the SJ.

Further studies are needed to investigate the effect of partial sleep deprivation on jump performance. It is necessary to determine how different amounts of partial sleep deprivation affect jump performance, whether there is a dose–response relationship between the magnitude (in hours per day and days per week) of partial sleep deprivation and jump performance, and which mechanisms contribute to the negative effect of sleep deprivation on jump performance. This study could not evaluate this directly because performance was not assessed after the first night, and the between-group comparison was not significant. Coaches may consider monitoring sleep duration before high-intensity power training, as two nights of severe sleep restriction (<4 h/night), compared with mild restriction, may be associated with reduced SJ performance.

## Figures and Tables

**Table 1:** Sleep duration (h) on Night 1 and Night 2 in the control (mild restriction) and experimental (severe restriction) groups.

	Sleep n1		Sleep n2	
	g1	g2	g1	g2
Mean	7.31	3.91	6.55	3.96
Std. Dev	1.26	0.24	0.83	0.27
Minimum	5.75	3.40	5.02	3.59
Maximum	9.50	4.18	7.72	4.69

Legend: g1 = control group; g2 = experimental group; sleep n1 = amount of sleep on the first night of the study protocol; sleep n2 = amount of sleep on the second night of the study protocol.

**Table 2:** Squat jump (SJ) and countermovement jump (CMJ) height (cm) at baseline (pre) and after Night 2 (post) in the control and experimental groups.

	SJ1		SJ2		CMJ1		CMJ2	
	g1	g2	g1	g2	g1	g2	g1	g2
Mean	34.09	32.63	34.82	31.18	36.71	35.47	36.73	34.58
Std. Dev	2.84	3.73	3.97	3.71	3.35	3.00	4.41	2.78
Minimum	28.80	25.20	30.80	23.80	31.10	30.70	31.10	29.90
Maximum	38.70	36.40	42.70	35.60	40.40	39.50	43.70	39.00

Legend: g1 = control group; g2 = experimental group; SJ = squat jump (1 = pre; 2 = post); CMJ = countermovement jump (1 = pre; 2 = post).

**Table 3:** Within-group pre–post changes in SJ and CMJ height (paired-samples *t*-tests).

	Mean Difference	Std	95% CI	t-Value	*p*-Value	Cohen’s d
SJ (g1)	0.73	3.32	−1.50, 2.96	0.69	0.51	0.23
CMJ (g1)	0.02	2.22	−1.47, 1.51	0.03	0.98	0.01
SJ (g2)	−1.44	1.78	−2.57, −0.31	−2.78	0.02	−0.81
CMJ (g2)	−0.88	1.99	−2.14, 0.38	−1.54	0.15	−0.45

Legend: g1 = control group; g2 = experimental group; SJ = squat jump; CMJ = countermovement jump; Std = standard deviation, CI = confidence interval.

**Table 4:** Between-group comparison of post-test SJ and CMJ adjusted for sleep duration (Quade’s nonparametric ANCOVA).

	F-Value	*p*-Value	Partial η^2^
SJ	0.12	0.73	0.01
CMJ	0.01	0.92	0.00

Legend: Partial η^2^ = effect size.

**Table 5:** Sensitivity analysis: Spearman correlations between total sleep duration (Night 1 + Night 2) and changes in jump performance (ΔSJ, ΔCMJ).

	Spearman r	*p*
TS vs. ∆SJ	0.26	0.25
TS vs. ∆CMJ	−0.09	0.68

Legend: TS = total sleep duration; ΔSJ = change in squat jump height (post–pre); ΔCMJ = change in countermovement jump height (post–pre); Spearman r = Spearman’s rank correlation coefficient; *p* = two-tailed *p*-value.

## Data Availability

The original contributions presented in the study are included in the article; further inquiries can be directed to the corresponding author.
